# Overexpression of Swedish mutant APP in aged astrocytes attenuates excitatory synaptic transmission

**DOI:** 10.14814/phy2.12665

**Published:** 2016-01-05

**Authors:** Shutaro Katsurabayashi, Hiroyuki Kawano, Miyuki Ii, Sachiko Nakano, Chihiro Tatsumi, Kaori Kubota, Kotaro Takasaki, Kenichi Mishima, Michihiro Fujiwara, Katsunori Iwasaki

**Affiliations:** ^1^ Department of NeuropharmacologyFaculty of Pharmaceutical SciencesFukuoka UniversityFukuokaJapan; ^2^ A.I.G. Collaborative Research Institute for Aging and Brain SciencesFukuoka UniversityFukuokaJapan

**Keywords:** Aging, alzheimer's disease, amyloid, astrocyte, synaptic release

## Abstract

Amyloid precursor protein (APP), a type I transmembrane protein, has different aspects, namely, performs essential physiological functions and produces *β*‐amyloid peptide (A*β*). Overexpression of neuronal APP is responsible for synaptic dysfunction. In the central nervous system, astrocytes – a major glial cell type – have an important role in the regulation of synaptic transmission. Although APP is expressed in astrocytes, it remains unclear whether astrocytic overexpression of mutant APP affects synaptic transmission. In this study, the effect of astrocytic overexpression of a mutant APP on the excitatory synaptic transmission was investigated using coculture system of the transgenic (Tg) cortical astrocytes that express the human APP695 polypeptide with the double mutation K670N + M671L found in a large Swedish family with early onset Alzheimer's disease, and wild‐type hippocampal neuron. Significant secretion of A*β* 1–40 and 1–42 was observed in cultured cortical astrocytes from the Tg2576 transgenic mouse that genetically overexpresses Swedish mutant APP. Under the condition, Tg astrocytes did not affect excitatory synaptic transmission of cocultured wild‐type neurons. However, aged Tg astrocytes cultured for 9 weeks elicited a significant decrease in excitatory synaptic transmission in cocultured neurons. Moreover, a reduction in the number of readily releasable synaptic vesicles accompanied a decrease in the number of excitatory synapses in neurons cocultured with aged Tg astrocytes. These observations indicate that astrocytic expression of the mutant APP is involved in the downregulation of synaptic transmission with age.

## Introduction

Alzheimer's disease (AD) is a neurodegenerative disorder accompanied by progressive memory deterioration. Synaptic dysfunction likely represents a major hallmark of the early stage of AD (Selkoe 2002; Haass and Selkoe 2007). In particular, progressive loss of synapses aggravates memory impairment before the formation of senile plaques. Morphological abnormalities such as the loss of synapses, deposition of senile plaques, and changes in fibrillogenesis are major characteristics of the AD brain. There are three major hypotheses regarding the mechanism assumed to be the target for therapeutic drugs for AD: (1) the cholinergic hypothesis, (2) the amyloid hypothesis, and (3) the tau hypothesis. A cholinesterase inhibitor and an NMDA receptor antagonist are drugs clinically used to inhibit the progression of AD‐type dementia (Rafii and Aisen 2015). On the other hand, the amyloid hypothesis has played a prominent role in explaining the pathology of AD. Recently, clinical trials have been envisaged on the basis of the A*β* hypothesis regarding the drug targeting components of the amyloid pathway using passive/active antiamyloid immunotherapy (Schenk et al. 1999; Muhs et al. 2007; Ryan and Grundman 2009; Lemere and Masliah 2010; Winblad et al. 2012) and an inhibitor of the *β*‐secretase cleaving enzyme (BACE)1 and BACE2 (Menting and Claassen 2014). In addition to the amyloid hypothesis, therapies targeting tau have been considered in immunotherapy based on the tau hypothesis (Lewis and Dickson 2015; Pedersen and Sigurdsson 2015; Sigurdsson 2016).

Amyloid precursor protein (APP), a type I transmembrane protein, is a fundamental element for production of the amyloid *β* (A*β*) peptide (Goedert 1987). Hence, aggregation of A*β* is accelerated when mutated APP is progressively cleaved by two aspartyl proteases, *β*‐ and *γ*‐secretases (Selkoe 2000). Aggregation of A*β* is the typical pathological feature of AD. Because APP is abundantly expressed in neurons (Selkoe et al. 1988; Mita et al. 1989), mutations of APP are responsible for abnormal synaptogenesis and synaptic dysfunction. In addition, exogenous administration of A*β* 1–42 causes attenuation of the long‐term potentiation, resulting from a decrease in the protein kinase A (PKA) activity (Vitolo et al. 2002). A*β* also causes a postsynaptic silencing through a selective downregulation of *α*‐amino‐3‐hydroxy‐5‐methyl‐4‐isoxazolepropionic acid (AMPA) receptors in hippocampal neuronal culture overexpressing human APP mutant (Ting et al. 2007).

On the other hand, some relevant functions of the astrocyte, a major glial cell type, were demonstrated recently: there is evidence of astrocytes' capacity for clearance of A*β*, where astrocytes migrate to the senile plaques in AD brain (Alarcon et al. 2005; Pihlaja et al. 2008). Because APP is expressed in astrocytes (Berkenbosch et al. 1990; Haass et al. 1991), it is essential to understand whether mutant astrocytes can release A*β*. If this is the case, this property of astrocytes can influence synapse formation and/or synaptic transmission.

Therefore, we hypothesized that astrocytic overexpression of mutated APP could produce A*β* and would affect the synaptic transmission. The purpose of this study was to confirm the existence of mutant APP in the Tg2576 transgenic astrocytes and to elucidate the functional roles of astrocytic mutant APP in synaptic transmission. We used primary autaptic culture of a single wild‐type (WT) murine hippocampal neuron placed on a microisland of cultured cortical astrocytes from Tg2576 heterozygous mice. This transgenic mouse is a familial AD model that expresses the human APP695 polypeptide with the double mutation K670N + M671L found in a large Swedish family with early onset AD (Hsiao et al. 1996; Kawarabayashi et al. 2001). Here, we show that aged cortical astrocytes from Tg2576 heterozygous mice attenuate synaptic transmission of cocultured hippocampal neurons, and this effect is dependent on the duration of culture (on age) of the transgenic astrocytes.

## Materials and Methods

### Animals

Swedish APP heterozygous male and B6 wild‐type female of the Tg2576 mice were purchased from Taconic Farms, Inc. (Hudson, NY). Cells for primary culture were obtained from newborn mice after decapitation under ether anesthesia, and every effort was made to minimize pain (see the Conflict of Interest below).

### Astrocyte culture

To obtain newborn Tg2576 mutant mice, we concluded a limited academic research breeding agreement on Tg2576 mice with Taconic Farms, Inc. Cultures of cerebral cortical astrocytes were prepared as reported previously (Bekkers and Stevens 1991; Burgalossi et al. 2012; Kawano et al. 2012). The details are as follows. Cerebral cortices of newborn Tg2576 mice were removed from brains in cold Hank's balanced saline solution (Wako, Japan) and the cells were dissociated with 0.05% trypsin–EDTA (Wako, Japan). The cells were then plated in the plating medium composed of Dulbecco's Modified Eagle Medium with GlutaMAX‐I and pyruvate (DMEM, Invitrogen, Carlsbad, CA) supplemented with 10% fetal bovine serum (FBS, Invitrogen) and 0.1% MITO+ Serum Extender (BD Biosciences, Franklin Lakes, NJ) in a 75 cm^2^ culture flask (250 mL, BD falcon, Franklin Lakes, NJ). The next day, the culture flask was gently rinsed once with a fresh plating medium to remove loosely attached cells. When the culture reached near‐confluence 2 weeks later, microglia and other small cells were removed by tapping the culture flask several times. The medium was replaced with a fresh plating medium, at which point the antimitotic agents 5‐fluoro‐2′‐deoxyuridine (8 *μ*mol/L, Sigma–Aldrich, St. Louis, MO) and uridine (20 *μ*mol/L, Sigma–Aldrich) were added to suppress glial proliferation and to maintain purity of the cell population. Some culture flasks were incubated for additional 4 weeks, with the plating medium containing the antimitotic agents replaced every week. This is because astrocytes cultured long‐term show progressive aging features in the presence of antimitotic agents in vitro (Kawano et al. 2012). The aged astrocyte culture (free of neurons) was used for experiments depicted in Figures 3, 4 and 5.

Adherent cells cultured in the culture flasks for 2 or 6 weeks were trypsinized and plated on either the plates prepared for large‐scale culture, at a density of 26,000 cells/cm^2^, or on the dot‐stamped coverslips prepared for microisland culture, at a density of 6000 cells/cm^2^. In both cases, the cells were cultured in a plating medium without antimitotic agents. When the astrocytes formed a monolayer (within a week), antimitotic agents were added again to the plating medium to curb further glial proliferation.

For large‐scale culture of astrocytes, sterilized 22‐mm coverslips (thickness No. 1, Matsunami, Japan) were treated with a 1:1 mixture of rat tail collagen (final concentration of 1 mg/mL, BD Biosciences) and poly‐D‐lysine (final concentration of 0.5 mg/mL, Sigma–Aldrich), with uniform spreading using a cotton swab. For the microisland culture of astrocytes, the coverslips were precoated uniformly with 0.5% liquefied agarose to prevent cells from attaching. The next day, a mixture of collagen and poly‐D‐lysine was applied on top of the agarose layer, using a custom‐made dot stamp designed to deposit a substrate in 300‐*μ*m squares with 500‐*μ*m edge‐to‐edge intervals.

### Autaptic single‐neuron culture

Hippocampal neurons were obtained from the brains of newborn ICR mice (Kyudo, Japan), and enzymatically dissociated in DMEM containing papain (2 U/mL, Worthington), for 60 min at 37°C. The cells were plated at a density of 1500 cells/cm^2^, onto the Tg2576 astrocyte microislands. Before the dissociated hippocampal neurons were plated, the conditioned medium of the astrocyte microisland culture was replaced with the Neurobasal‐A medium (Invitrogen) containing 2% B‐27 supplement (Invitrogen) and 1% GlutaMAX‐I supplement (Invitrogen). Hippocampal single neurons were then cocultured for 13–17 days in vitro (DIV) on microisland astrocytes that had already been cultured for different periods (3 or 7 weeks). Hence, the astrocytes had eventually been cultured for 5 or 9 weeks by the time of electrophysiological recordings. Former autaptic neurons cocultured with young astrocytes were used for experiments shown in Figure 2, and later autaptic neurons cocultured with aged astrocytes were used for experiments depicted in Figures 3, 4, and 5.

### RNA extraction and RT‐PCR

Total RNA was extracted from cultured astrocytes using the TRIzol reagent (Invitrogen) according to the manufacturer's instructions. RNA (0.5 *μ*g) from each sample was reverse‐transcribed using the ReverTra AceR qPCR RT Kit (TOYOBO, Osaka, Japan) according to the manufacturer's protocol. The resulting cDNA was amplified using the following thermal parameters: denaturation at 94°C for 10 min followed by 20 cycles of 94°C for 30 sec, 55°C for 30 s, and 72°C for 1 min. The following primers were used: GFAP forward 5′‐TCCTGGAACAGCAAAACAAG‐3′, reverse 5′‐CAGCCTCAGGTTGGTTTCAT‐3′; CD11b forward 5′‐CTCCGGTAGCATCAACAACAT‐3′, reverse 5′‐TGATCTTGGGCTAGGGTTTCT‐3′; MAP2 forward 5′‐TCCCCAGCTACTCCTAAGCA‐3′, reverse 5′‐AGAGCCACATTTGGATGTCA‐3′; and *GAPDH* forward 5′‐GCTGCCAAGGCTGTGGGCAAG‐3′, reverse 5′‐GCCTGCTTCACCACCTTC‐3′. The PCR products were analyzed by electrophoresis on 2% agarose gels, which were stained with ethidium bromide and visualized using FluorChem8900 (Alpha Innotech, Santa Clara, CA).

### Quantitative RT‐PCR analysis

The cDNA was amplified using FastStart Essential DNA Green Master (Roche Diagnostics, Mannheim, Germany) on a LightCycler Nano (Roche Diagnostics) with the following primer sets: APP forward 5′‐CATCCAGAACTGGTGCAAGCGG‐3′, reverse 5′‐GACGGTGTGCCAGTGAAGATG‐3′ (human APP: NM_201414, mouse APP: NM_007471.2); IL6 forward 5′‐GCCTTCCCTACTTCACAAGTC‐3′, reverse 5′‐CAGAATTGCCATTGCACAACTC‐3′; and *GAPDH* forward 5′‐GCTGCCAAGGCTGTGGGCAAG‐3′, reverse 5′‐GCCTGCTTCACCACCTTC‐3′. The PCR cycling parameters were denaturation at 95°C for 10 min followed by 40 cycles of 95°C for 10 s, 60°C for 10 sec, and 72°C for 15 s. Relative mRNA expression was determined using the 2^−ΔΔCT^ method, with standardization against *GAPDH* and normalization to young astrocytes.

### Western blotting

Whole‐cell protein lysates were collected from the plates containing the large‐scale culture of astrocytes using PRO‐PREP Protein Extraction Solution (iNtRON Biotechnology, Seongnam‐Si, Korea). The total protein concentration of each cell lysate was determined using Pierce BCA Protein Assay Kit (Thermo Scientific, Waltham, MA). Equivalent amounts of each cell extract were separated using SDS‐PAGE and transferred to PVDF membranes according to standard procedures. After blocking with 5% skim milk in TBST (20 mmol/L Tris, pH 7.4, 65 mmol/L NaCl, 0.1% Tween 20), membranes were probed with primary antibodies (anti‐APP, 1:1,000; R&D Systems, anti–*β*‐actin, 1:2,000; Abcam, Cambridge, UK) for 1 h at room temperature. The membranes were washed thrice with TBST and incubated with a horseradish peroxidase (HRP)–conjugated secondary antibody (1:10,000, Santa Cruz Biotechnology, Dallas, TX) for 1 h at room temperature. After washing thrice with TBST, the bands were developed using ECL Advance Western Blotting Detection Kit (GE healthcare, Little Chalfont, UK) and detected using FluorChem8900 (Alpha Innotech; San Leandro, CA). Densitometric analysis was performed using the ImageJ software (1.46j, Wayne Rasband, NIH).

### Quantification of Aβ in the astrocyte‐conditioned medium

For the detection of A*β* 1–40 and A*β* 1–42 secreted from the cultured astrocytes, the astrocyte‐conditioned medium (ACM) was collected from the plates containing the large‐scale culture of astrocytes incubated for 5 weeks. Then, ACM was analyzed using sensitive sandwich ELISA with either the Human amyloid *β* (1–40) Assay kit or the Human amyloid *β* (1–42) Assay kit (IBL, Gunma, Japan), according to the instructions of the manufacturer. The A*β* quantities were calculated by comparing the absorbance obtained from duplicate samples to standard curves of either A*β* 1–40 or A*β* 1–42.

### Autaptic culture electrophysiology

Microislands containing a single neuron forming recurrent synapses (autapses) were used for synaptic recordings. The recordings were performed on neuronal 13–17 DIV to ensure that synaptic responses were stable with reliable space clamping. Excitatory postsynaptic currents (EPSCs) were recorded using a patch clamp amplifier (MultiClamp 700B, Molecular Devices), in the whole‐cell configuration in the voltage clamp mode, at a holding potential (V_h_) of −70 mV, and at room temperature in all cases. Patch pipette resistance was 4–5 MΩ, and 70–80% of access resistance was compensated. Autaptic neurons showed synaptic transmission in response to an action potential elicited by a brief (2 ms) somatic depolarization pulse (to +0 mV) from the patch pipette. Because hypertonic sucrose forces the release of all synaptic vesicles in the readily releasable pool (RRP) (Rosenmund and Stevens 1996), the number of synaptic vesicles in RRP was calculated by dividing an RRP charge by the averaged charge of spontaneous miniature excitatory postsynaptic currents (mEPSCs) in the same neuron (Kawano et al. 2012). mEPSCs were recorded in the presence of the Na^+^ channel blocker tetrodotoxin (TTX, 0.5 *μ*mol/L). Vesicular release probability (P_vr_) was defined as the probability of release of individual synaptic vesicles in response to an action potential. P_vr_ was calculated by dividing the action potential–induced EPSC charge by the sucrose‐induced transient EPSC charge. The synaptic responses were recorded at a sampling rate of 20 kHz and were filtered at 10 kHz. Data were excluded from analysis if a leak current of >300 pA was observed. The data were analyzed offline using the AxoGraph X 1.2 software (AxoGraph Scientific, Berkeley, CA). mEPSCs with an amplitude threshold of 5 pA were detectable.

### ß‐Galactosidase staining

Astrocytes mass cultured on coverslips were washed twice with phosphate‐buffered saline (PBS) and fixed in PBS containing 4% paraformaldehyde for 20 min at room temperature. Senescence in astrocytic cultures was assessed using ß‐galactosidase–based Senescence Cells Staining Kit, according to the manufacturer's instructions (CS0030‐1KT, Sigma–Aldrich). Astrocytes stained at pH 6 (4 mmol/L potassium ferricyanide solution and 4 mmol/L potassium ferrocyanide solution) were visualized using a light microscope in bright‐field mode. The stained area corresponds to the amount of expression of *β*‐galactosidase. The images were obtained from three separate cultures.

### Immunocytochemistry

Autaptic hippocampal neurons were fixed in PBS containing 4% paraformaldehyde for 20 min at room temperature and blocked and permeabilized with PBS containing 5% normal goat serum and 0.1% Triton X‐100, for 30 min. After blocking, the samples were incubated overnight at 4°C with the following primary antibodies: an antimicrotubule‐associated protein 2 antibody (MAP 2, guinea pig polyclonal antiserum, Synaptic Systems, 1:1,000 dilution) and an antivesicular glutamate transporter 1 antibody (VGLUT 1, rabbit polyclonal, affinity purified, Synaptic Systems, 1:2000 dilution) in a humidity chamber. After washing three times with PBS for 5 min, autaptic neurons were incubated with appropriate species‐specific fluorophore‐conjugated goat secondary antibodies (Alexa Fluor 488 for MAP 2 and Alexa Fluor 594 for VGLUT 1, 1:400 dilutions, Invitrogen) for 30 min at room temperature. Then, autaptic neurons were washed more than three times with PBS for 5 min, and were mounted with ProLong^®^ Gold antifade mounting reagent (Invitrogen) on the slide glass.

### Image acquisition and quantification

Confocal images of autaptic neurons were obtained using an objective lens C‐Apochromat, 40 × , NA 1.2 (Carl Zeiss, Oberkochen, Germany), with sequential acquisition settings at high‐resolution settings (1024 × 1024 pixels) of the confocal microscope (LMS710, Carl Zeiss). The parameters of each image were optimized for the z‐stack setting (a depth of 9 *μ*m with 0.5 *μ*m steps) and a pinhole setting (1 Airy unit, 0.9 *μ*m). The depth range sufficiently covered the thickness of the neurites in our autaptic culture. The confocal microscope settings were the same for all scans in each experiment. A single autaptic neuron was selected for each scan in a blinded manner, based on MAP 2 fluorescence images.

The sizes and numbers of synaptic puncta were detected using the ImageJ software. The procedures used to analyze synaptic puncta were published previously (Kakazu et al. 2012; Kawano et al. 2012; Iwabuchi et al. 2014). Background noise was removed by subtracting Gaussian filtered signals from the original image (Iwabuchi et al. 2014). The processed images were then binarized using threshold at top 0.01% of cumulative intensity of background region for VGLUT1images. A punctum was selected with a size threshold greater than or equal to nine pixels.

### Solutions

The standard extracellular solution for synaptic recordings was (in mmol/L) NaCl 140, KCl 2.4, HEPES 10, glucose 10, CaCl_2_ 2, MgCl_2_ 1, pH 7.4, with adjusted osmotic pressure of 315–320 mOsm. Patch pipettes were filled with an intracellular solution composed of (in mmol/L) potassium gluconate 146.3, MgCl_2_ 0.6, ATP‐Na_2_ 4, GTP‐Na_2_ 0.3, phosphocreatine 12, EGTA 1, HEPES 17.8 (pH 7.4), creatine phosphokinase 50 U/mL. Hypertonic solutions for determining the RRP size were prepared by adding 0.5mol/L sucrose to the standard extracellular solution. The extracellular solutions were applied using a fast‐flow application system (SF‐77B, Warner Instruments). Each flow pipe had a large diameter (430 *μ*m), ensuring that the solution was applied to all parts of an autaptic neuron on an astrocytic microisland (300 × 300 *μ*m). This configuration is necessary if the addition of sucrose or TTX is to induce synaptic responses from all nerve terminals of the neuron being recorded. All chemicals were purchased from Sigma–Aldrich, except where specified otherwise.

### Statistical analysis

Data were calculated as mean ± SEM. Analyses were performed using the nonparametric unpaired *t* test, and significance was assumed when *P *<* *0.05 in comparison with data from the WT group that had been cultured for the same length of time.

## Results

### Properties of the cultured Tg2576 astrocytes

**Figure 1:**
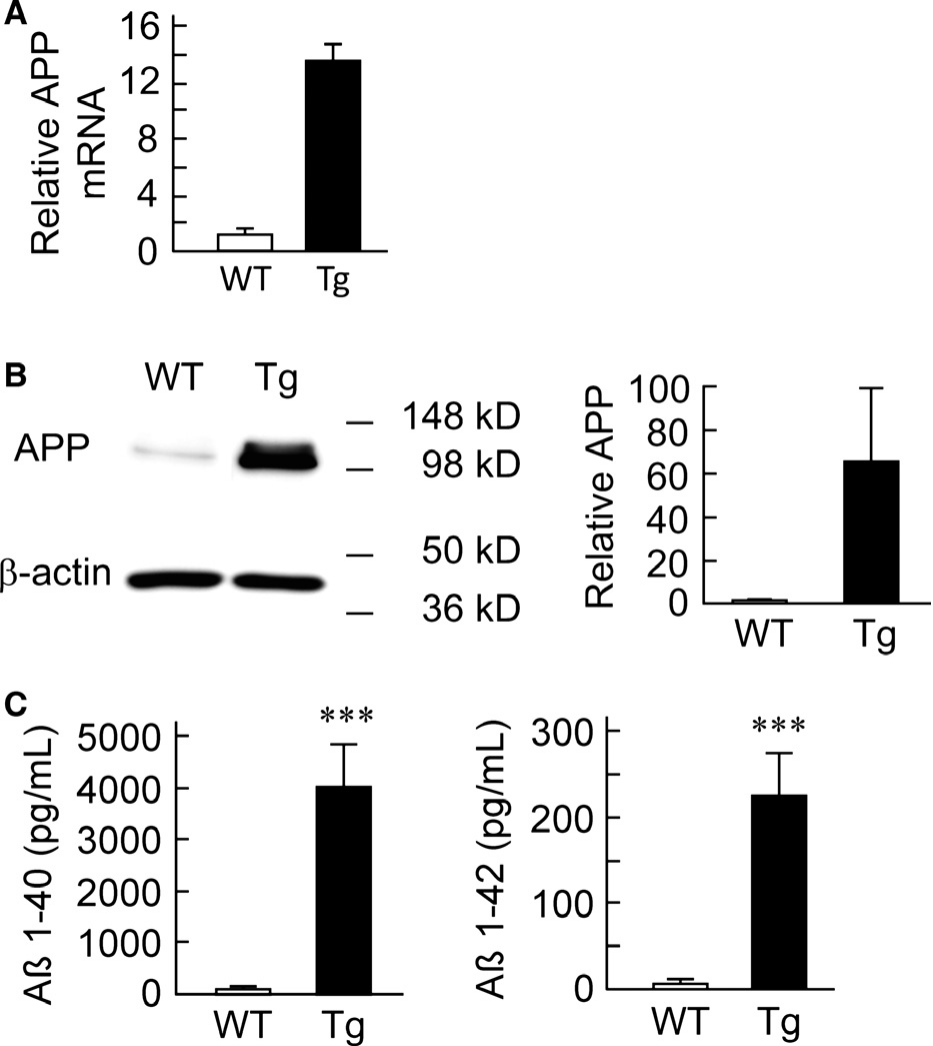
Amyloid precursor protein (APP) is expressed in cultured cortical astrocytes from wild‐type (WT) and transgenic (Tg) 2576 mice. (A) Relative APP mRNA levels in the astrocytes from WT (9 cultures) and Tg2576 mice (7 cultures). Note that APP mRNA was overexpressed in Tg2576 astrocyte culture. (B) Western blot analysis in WT (4 cultures) or Tg2576 (4 cultures) astrocytes. Note that APP was overexpressed in Tg2576 astrocyte culture. (C) Quantification of A*β* 1–40 (WT: 7 cultures, Tg: 7 cultures) and A*β* 1–42 (WT: 8 cultures, Tg: 8 cultures) in cultured WT or Tg astrocyte‐conditioned medium. The data are shown as mean ± SEM.

At the early stage of Alzheimer's disease (AD), soluble amyloid *β* (A*β*) is produced when *β*‐secretase and *γ*‐secretase cleave amyloid precursor protein (APP) (Selkoe 2000). It is known that APP is abundant in neurons (Goedert 1987; Selkoe et al. 1988; Mita et al. 1989), but is also expressed in astrocytes, a major glial cell type in the brain (Berkenbosch et al. 1990; Haass et al. 1991). To understand the properties of astrocytes expressing mutant APP, we utilized Tg2576 mouse strain that expresses mutant human APP695 (Hsiao et al. 1996; Kawarabayashi et al. 2001). WT and Tg cortical cell cultures were prepared from newborn Tg2576 mice. Our large‐scale cultures expressed glial fibrillary acidic protein (GFAP) mRNA, but did not express mRNA of either microtubule‐associated protein 2 (MAP2) or CD11b, suggesting that astrocyte population was pure and the extraction procedure was effective and did not cause contamination with neurons or microglia (data not shown). As shown in Figure 1A, the Tg astrocytes overexpressed APP mRNA by 12‐fold compared with the WT astrocytes (WT: 9 cultures, Tg: 7 cultures). Western blot analysis showed that the astrocytic APP protein expression was 60‐fold higher in Tg astrocytes on average (WT: 4 cultures, Tg: 4 cultures; Figure 1B). Because mutant APP was markedly overexpressed in the Tg astrocytes, the Tg astrocytes may also produce large amounts of A*β*. To confirm this supposition, we measured concentrations of A*β* 1–40 and A*β* 1–42 in the astrocyte‐conditioned medium (ACM) using sandwich ELISA. WT astrocytes did not produce detectable amounts of A*β*, but Tg astrocytes released significant quantities of A*β* 1–40 (WT, 88.9 ± 19.5 pg/mL*,* 7 cultures, Tg: 3,994.7 ± 780.4 pg/mL, 7 cultures) and A*β* 1–42 (WT: 4.8 ± 3.3 pg/mL*,* 8 cultures, Tg: 222.0 ± 49.4 pg/mL, 8 cultures) in ACM (Fig. 1C). The concentration of A*β* 1–40 was somehow 18‐fold higher than that of A*β* 1–42 in the culture supernatant of Tg astrocytes.

### Overexpression of astrocytic mutant APP does not affect the excitatory synaptic transmission

Addition of exogenous A*β* to neurons affects the excitatory synaptic transmission (Parodi et al. 2010; Yao et al. 2013). The effect of addition of exogenous A*β* is strongly dependent on the concentration of A*β* and/or the exposure time (Parodi et al. 2010). Because astrocytes have an important role in the information processing by synapses of the central nervous system (Allen and Barres 2009; Eroglu and Barres 2010), we speculated that the synaptic transmission may be attenuated by chronic exposure to A*β* 1–40 and 1–42 released from the Tg astrocytes. Therefore, we analyzed the effects of overexpression of astrocytic mutant APP on the basal synaptic transmission using a coculture system of Tg astrocytes and WT neurons. To assess the synaptic transmission, hippocampal neurons obtained from newborn ICR mice (wild‐type) were plated on microislands of cultured astrocytes that had been isolated from Tg2576 mice. The autaptic primary culture is a well‐established model of the synapse in vitro, which involves synapses with own dendrites and the soma of a neuron is planted within the microisland field of astrocytes (Bekkers and Stevens 1991; Burgalossi et al. 2012; Kawano et al. 2012). In order to ensure that the factors secreted from the surrounding astrocytes were present in the culture medium by the time of synaptic recordings; medium was not changed during neuronal culture. Then, we focused on AMPA‐mediated transmission of excitatory glutamatergic synapses in autaptic hippocampus neurons cultured with either WT or Tg astrocytes, and we compared these two conditions alongside.

**Figure 2:**
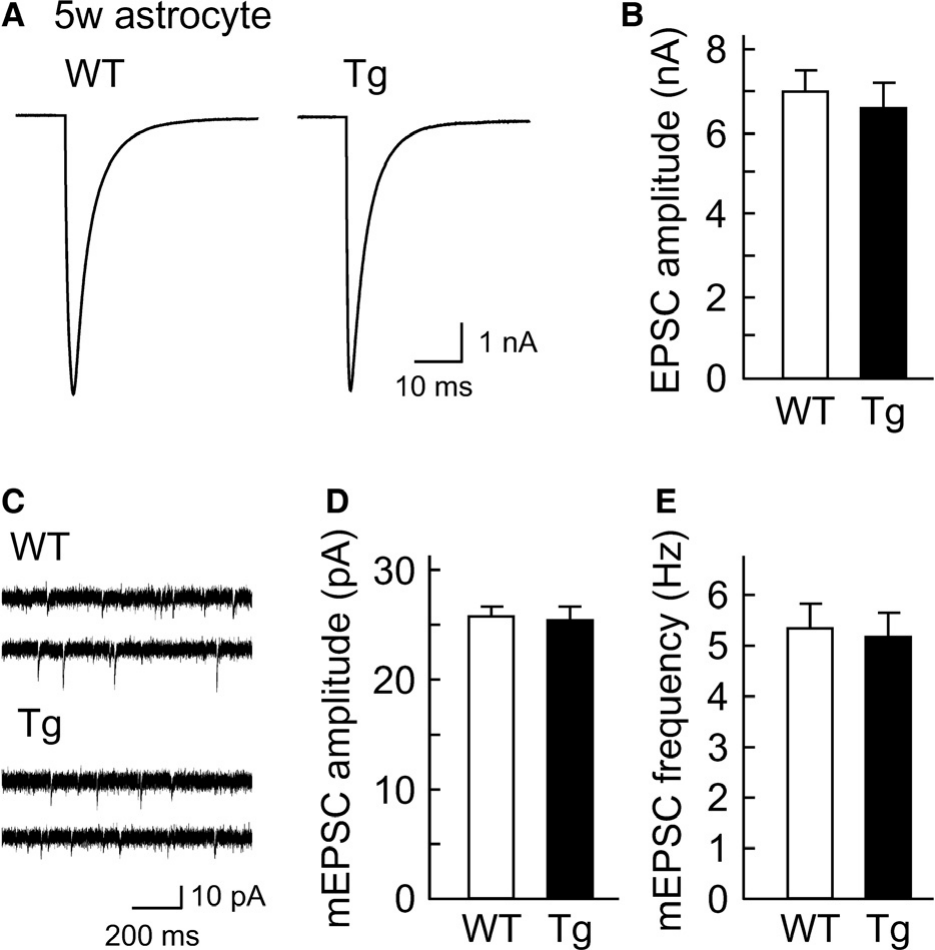
Excitatory synaptic transmission is not affected by the transgenic (Tg) astrocyte coculture. (A) Representative traces of evoked excitatory postsynaptic currents (EPSCs) recorded from autaptic hippocampal neurons cocultured with wild‐type (WT) or Tg astrocytes. Depolarization artifacts caused by the generated action currents have been removed for clarity of presentation. (B) Average amplitudes of the evoked EPSCs in neurons cocultured with either WT (*n *= 176/10 cultures) or Tg astrocytes (*n *= 177/10 cultures). (C) Representative mEPSC traces in neurons cocultured with WT or Tg astrocytes. (D) An averaged mEPSC amplitude in neurons cocultured with either WT (*n *= 176/10 cultures) or Tg astrocytes (*n *= 176/10 cultures). (E) An averaged mEPSC frequency in neurons cocultured with either WT (*n *= 176/10 cultures) or Tg astrocytes (*n *= 176/10 cultures). The data are shown as mean  ± SEM.

The AMPA receptor‐mediated excitatory postsynaptic current (EPSC) was recorded (Fig. 2A) and was blocked by 10 *μ*M CNQX, a competitive antagonist of AMPA subtype of the ionotropic glutamate receptors (data not shown). Unexpectedly, the evoked EPSC amplitude was almost unchanged in neurons cultured with Tg astrocytes, relative to that of control where a neuron was cocultured with WT astrocytes (WT: 6.8 ± 0.5 nA, *n *=* *176/10 cultures, Tg: 6.5 ± 0.5 nA, *n *=* *177/10 cultures; Fig. 2B).

Spontaneous miniature EPSCs (mEPSCs) were recorded in the same preparations (Fig. 2C). The mEPSC events correspond to the activation of postsynaptic AMPA receptors by the amount of a neurotransmitter released from a single synaptic vesicle (Katz and Miledi 1979; Bekkers and Stevens 1995). As a result, the amplitude of mEPSCs (WT: 25.3 ± 0.6 pA, *n *=* *176/10 cultures; Tg: 25.2 ± 0.6 pA, *n *=* *176/10 cultures; Fig. 2D) or the frequency of mEPSCs (WT: 5.3 ± 0.4 Hz, *n *=* *176/10 cultures; Tg: 5.1 ± 0.4 Hz, *n *=* *176/10 cultures; Fig. 2E) was not changed between neurons cocultured with WT and Tg astrocytes. These data indicate that AMPA‐mediated excitatory synaptic transmission remains unchanged during neuronal culture with the short‐term culture of Tg astrocytes overexpressing mutant APP.

### Long‐term culture of astrocytes from Tg2576 mice shows aging signs in vitro

**Figure 3:**
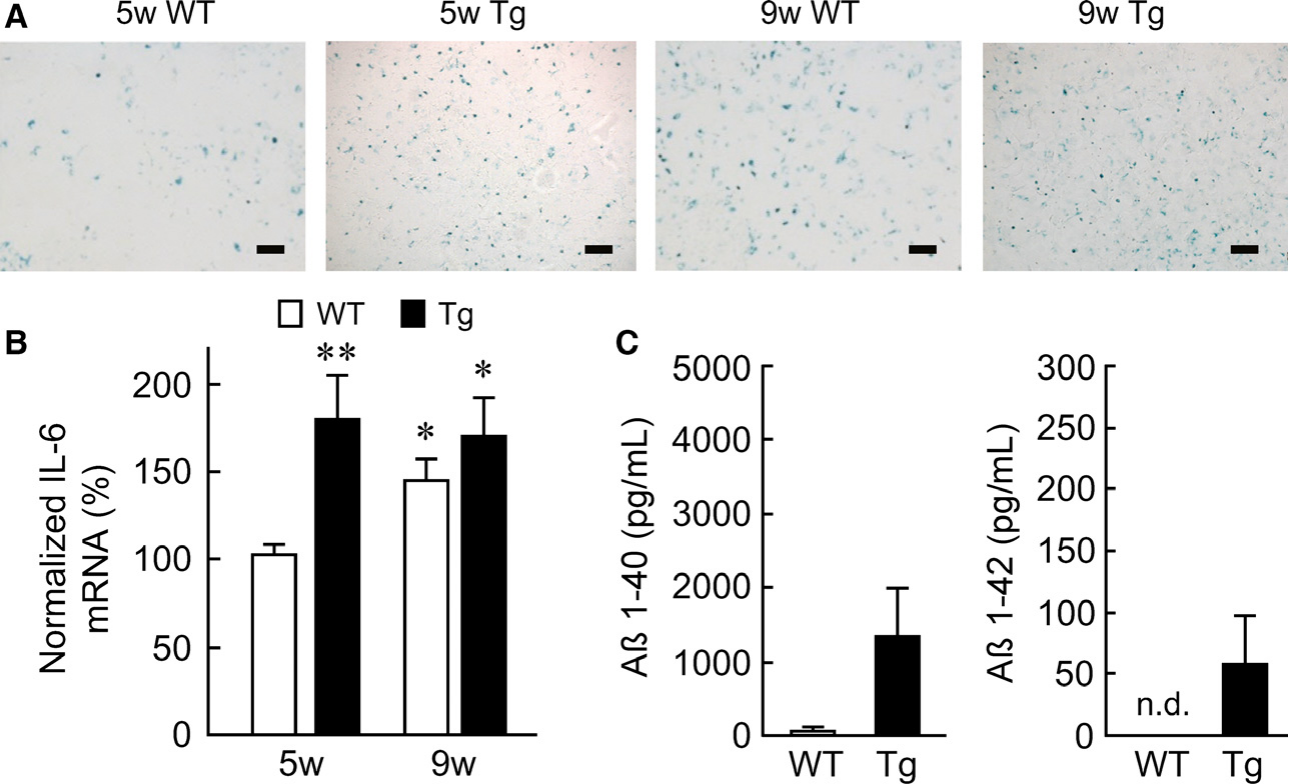
Astrocytes cultured for long periods show detectable signs of aging. (A) Representative bright‐field images of WT and Tg2576 astrocytes mass‐cultured for 5 or 9 weeks in vitro. Scale bars represent 200 *μ*m. (B) Normalized IL‐6 mRNA expression in large‐scale WT and Tg2576 astrocytes cultured for 5 or 9 weeks in vitro (8 cultures at each age). *P < 0.05, **P < 0.01 versus 5‐week WT astrocytes. The data are shown as mean ± SEM. (C) Quantification of A*β* 1–40 (WT: 5 cultures; Tg: 5 cultures) and A*β* 1–42 (WT: 5 cultures; Tg: 5 cultures) in the culture medium of aged WT or aged Tg astrocyte‐conditioned culture medium. The data are shown as mean ± SEM.

The capacity for A*β* clearance gradually declines as a result of the progression of AD and aging (Gitter et al. 1995; Hu et al. 1998). This indicates that cellular aging would be one of the risk factors for the development of AD. Thus, the fact that Tg astrocytes did not produce any reduction in synaptic activity may be due to young astrocytes' sufficient ability to remove A*β*. We recently reported that a long‐term culture (>9 weeks) of astrocytes exhibited the features of aging in vitro (Kawano et al. 2012). Accordingly, WT and Tg astrocytes were cultured for 9 weeks in the following experiments. Astrocytes in the large‐scale culture (free of neurons) were assessed for endogenous *β*‐galactosidase activity as a function of aging (van der Loo et al. 1998; Pertusa et al. 2007; Kawano et al. 2012). As mentioned above, the senescence was significantly higher in both the WT and the Tg astrocytes that had been cultured for 9 weeks compared to that in 5‐week‐old cells (Fig. 3A). The expression of IL‐6 mRNA increases significantly in aged astrocytes (Bhat et al. 2012), and therefore, we also measured and compared the IL‐6 mRNA expression between 5‐ and 9‐week‐old cultures as an alternative assay for the determination of astrocyte aging. The 9‐week‐old WT and Tg astrocytes both exhibited a significant increase in IL‐6 mRNA expression compared with the 5‐week‐old WT astrocytes (WT: 8 cultures, Tg: 8 cultures; Fig. 3B). These indicate that the long‐term culture (>9 weeks) of Tg2576 astrocytes promises signs of aging in vitro. Notably, the expression of IL‐6 mRNA was higher in the Tg astrocytes cultured for 5 weeks than in the 5‐week‐old WT astrocytes (Fig. 3B).

We next measured concentrations of A*β* 1–40 and A*β* 1–42 in ACM obtained from the long‐term culture of WT and Tg astrocytes. Aged WT astrocytes did not produce detectable amounts of A*β*, but aged Tg astrocytes released higher amounts of A*β* 1–40 (WT: 37.2 ± 22.1 pg/mL, 5 cultures; Tg: 1,312.4 ± 644.85 pg/mL, 5 cultures) and A*β* 1–42 (WT: not detectable, 5 cultures; Tg: 55.46 ± 39.55 pg/mL, 5 cultures) in ACM (Fig. 3C). Notably, the concentrations of A*β* 1–40 and A*β* 1–42 from the long‐term culture of Tg astrocytes were much lower than those from the short‐term culture of Tg astrocytes (Fig. 1C).

### Excitatory synaptic transmission is attenuated by aged Tg astrocytes overexpressing mutant APP

**Figure 4:**
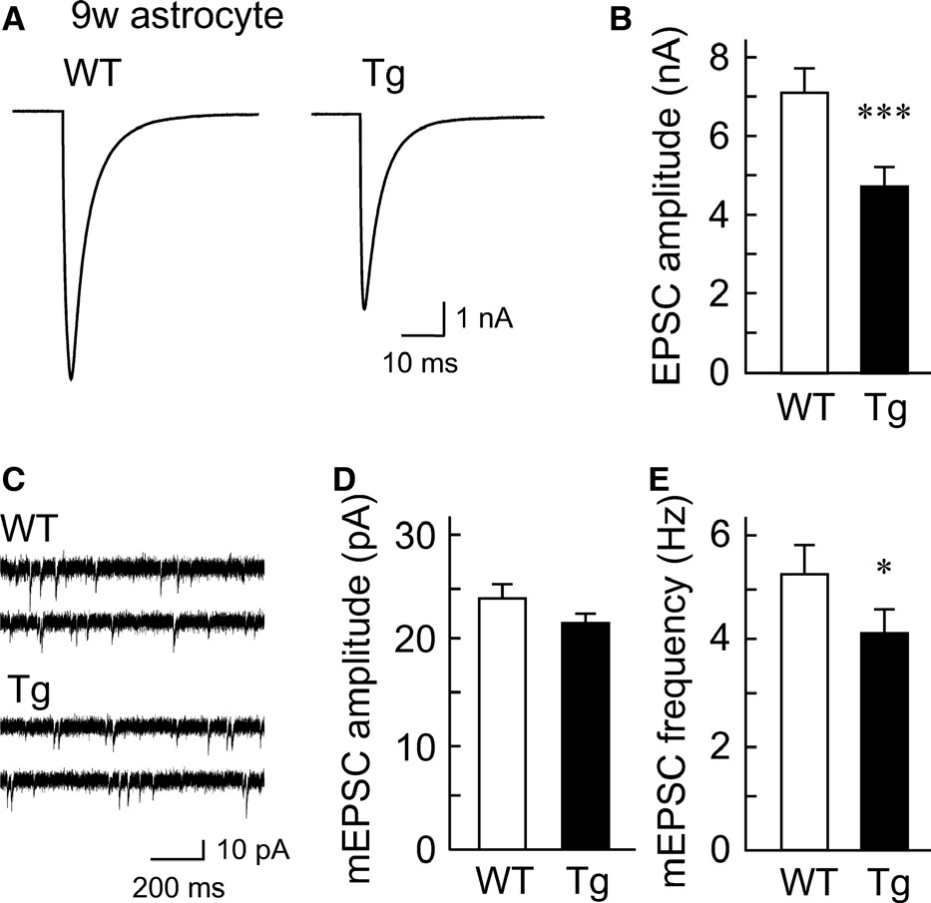
Excitatory synaptic transmission is attenuated in coculture with aged transgenic (Tg) astrocytes. (A) Representative traces of evoked excitatory postsynaptic currents (EPSCs) recorded from a single autaptic hippocampal neuron cocultured with either wild‐type (WT) or Tg astrocytes that had been cultured for 9 weeks. Depolarization artifacts caused by the generated action currents have been removed for clarity of presentation. (B) Average amplitudes of the evoked EPSCs in neurons cocultured with either aged WT (*n *= 139/9 cultures) or aged Tg astrocytes (*n *= 144/9 cultures). ***P < 0.001. (C) Representative mEPSC traces in neurons cocultured with either aged WT or aged Tg astrocytes. (D) An averaged mEPSC amplitude in neurons cocultured with either aged WT (*n *= 104/9 cultures) or aged Tg astrocytes (*n *= 120/9 cultures). (E) An averaged mEPSC frequency in neurons cocultured with either aged WT (*n *= 104/9 cultures) or aged Tg astrocytes (*n *= 120/9 cultures). *P <0.05. The data are shown as mean ±SEM.

To understand the effect of overexpression of mutant APP on synaptic transmission in condition of aged astrocytes, the evoked EPSCs were recorded in autaptic individual hippocampal neurons cocultured with aged WT and aged Tg astrocytes (Fig. 4A). The evoked EPSC amplitude was significantly decreased in neurons cultured with aged Tg astrocytes, relative to that of the control, where a neuron was cocultured with aged WT astrocytes (WT: 7.0 ± 0.5 nA, *n *=* *139/9 cultures, 95% confidence interval (CI) 6.1–7.9 nA; Tg: 4.6 ± 0.4 nA, *n *=* *144/9 cultures, 95% CI 3.9–5.4 nA; Fig. 4B). We then tested whether the decrease in the EPSC amplitude was due to a change in the presynaptic functions (Priller et al. 2007, 2009) and/or a decrease in the AMPA receptor density, such as internalization of receptors (Ting et al. 2007; Gu et al. 2009). Accordingly, mEPSCs were recorded (Fig. 4C). The amplitude of mEPSCs was not changed between neurons cocultured with aged WT or aged Tg astrocytes (WT: 23.6 ± 0.7 pA, *n *=* *104/9 cultures; Tg: 21.1 ± 0.7 pA, *n *=* *120/9 cultures; Fig. 4D). In contrast, the frequency of mEPSCs was significantly decreased in neurons cocultured with aged Tg astrocytes (WT: 5.2 ± 0.4 Hz, *n *=* *104/9 cultures, 95% CI 4.43–6.08 Hz; Tg: 4.1 ± 0.4 Hz, *n *=* *120/9 cultures, 95% CI 3.43–4.85 Hz; Fig. 4E). These data indicate that postsynaptic AMPA receptor density remains unchanged during neuronal culture in the presence of aged Tg astrocytes overexpressing mutant APP. Instead, a decrease in the action potential‐evoked EPSC amplitude must result from a change in the presynaptic release machinery and/or a decrease in the number of excitatory synapses.

**Figure 5:**
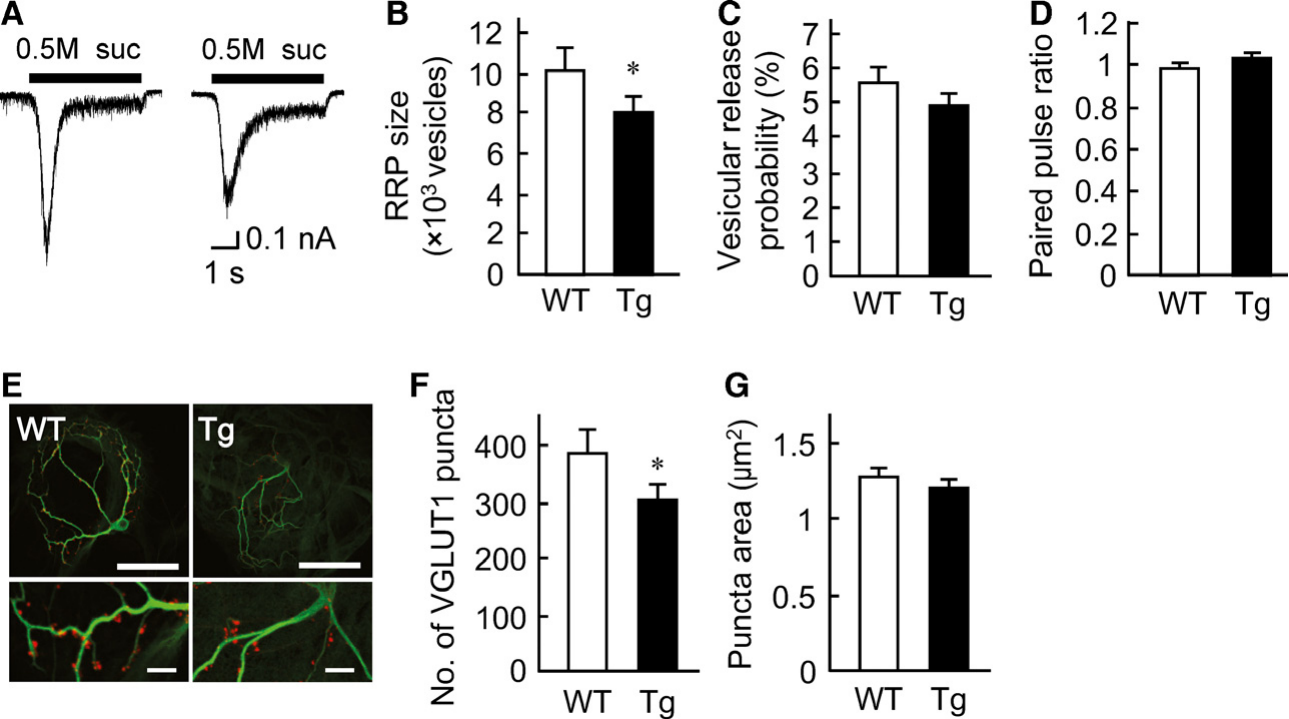
The number of excitatory synapses is decreased in coculture with aged transgenic (Tg) astrocytes. (A) Representative traces of response to 0.5 mol/L hypertonic sucrose solution (5 sec, black bar) in an autapse neuron cocultured with either the aged wild‐type (WT) or aged Tg astrocytes. (B) Number of synaptic vesicles in readily releasable pool of autaptic neurons cocultured with either aged WT (*n *= 103/9 cultures) or aged Tg astrocytes (*n *= 117/9 cultures). (C) P_vr_ in single autaptic neurons cocultured with either aged WT (*n *= 135/9 cultures) or aged Tg astrocytes (*n *= 138/9 cultures). (D) Paired pulse ratio (EPSC_2_/EPSC_1_) in single autaptic neurons cocultured with either aged WT (*n *= 83/5 cultures) or aged Tg astrocytes (*n *= 86/5 cultures). (E) Representative images of autaptic neurons immunostained for the excitatory nerve terminal marker VGLUT 1 (red) and the dendritic marker MAP 2 (green). Parts of images in the top row (scale bar 50 *μ*m) are enlarged in the bottom row (scale bar 5 *μ*m). (F) The number of VGLUT 1–positive synaptic puncta in autaptic neurons cocultured with either aged WT (*n *= 44/4 cultures) or aged Tg astrocytes (*n *= 50/4 cultures). (G) An averaged size of synaptic puncta labeled with VGLUT 1 in individual autaptic neurons. The data were obtained from the samples used in (F). The data are shown as mean ± SEM.

To confirm the presynaptic mechanisms in detail, we next measured the readily releasable pool of synaptic vesicles (RRP) in a single neuron cocultured with aged WT or aged Tg astrocytes (Fig. 5A). We found that the number of synaptic vesicles in the RRP was significantly decreased in neurons cocultured with aged Tg astrocytes (WT: 9984.0 ± 825.2 vesicles, *n *=* *103/9 cultures, 95% CI 8749.9–12096.7 vesicles; Tg: 7821.0 ± 630.7 vesicles, *n *=* *117/9 cultures, 95% CI 6705.0–9266.2 vesicles; Fig. 5B).

We next estimated the vesicular release probability (P_vr_). A fundamental property of P_vr_ is that action potential‐evoked release comes from a part of RRP that is affected by a hypertonic sucrose solution (Rosenmund and Stevens 1996). If a decrease in the evoked EPSC amplitude was due to a change in the release machinery, then P_vr_ may be altered. Nevertheless, P_vr_ was unchanged between the two groups (WT: 5.5 ± 0.3%, *n *=* *135/9 cultures; Tg: 4.8 ± 0.3%, *n *=* *138/9 cultures; Fig. 5C). Conversely, the change in presynaptic release probability is generally assessed by changes in the paired pulse ratio (PPR), defined as the ratio of the amplitude of the second EPSC to that of the first EPSC evoked by action potentials at short intervals (e.g., 50 ms). Therefore, we estimated PPR as another assessment of the release machinery. PPR did not differ between the two groups (WT: 0.97 ± 0.03, *n *=* *83/5 cultures; Tg: 1.02 ± 0.02, *n *=* *86/5 cultures; Fig. 5D). In summary, the lack of change in the Pvr or PPR implied that the release machinery was normal in neurons cocultured with aged Tg astrocytes.

The reduction in synaptic release without a change in the release machinery would be indeed evidence of a decrease in the RRP size. In other words, the RRP size was reduced without change in the vesicular release machinery in a single neuron cocultured with aged Tg astrocytes. As the application of sucrose was used to measure the total number of releasable vesicles in the RRP of single neurons (Rosenmund and Stevens 1996), the decrease in RRP size is reminiscent of the reduction in the number of synapses per neuron. If the number of synapses were unchanged, we would then conclude that the reduction in RRP size may be due to a reduction in transmitter concentration in the synaptic cleft. Accordingly, we counted the number of glutamatergic synapses in the autaptic hippocampal neurons. It is widely accepted that the majority of the excitatory synapses in autaptic hippocampal neurons contain vesicular glutamate transporter 1 (VGLUT 1); to be precise, the number of VGLUT 1‐staining puncta corresponds to the number of excitatory synapses (Wojcik et al. 2004; Li et al. 2013). We used an anti‐VGLUT 1 antibody as a marker for the glutamatergic nerve terminals in the immunocytochemical staining (red puncta, Fig. 5E). The number of puncta labeled as VGLUT 1‐positive was significantly decreased by the aged Tg astrocytes (WT: 381.5 ± 33.5, *n *=* *44/4 cultures, 95% CI 313.9–449.0; Tg: 295.5 ± 24.3, *n *=* *50/4 cultures, 95% CI 246.8–344.2; Fig. 5F). In contrast, the size of the VGLUT 1‐positive puncta was unchanged (WT: 1.25 ± 0.04 *μ*m^2^, *n *=* *44/4 cultures; Tg: 1.18 ± 0.03 *μ*m^2^, *n *=* *50/4 cultures; Fig. 5G). Because the tendency for a reduction in the number of VGLUT 1‐positive puncta was quite similar to that for the RRP size, we speculated that the decrease in the RRP size was mainly attributable to a decrease in the number of excitatory synapses. Hence, we consider that a reduction in neurotransmitter concentration in the synaptic cleft would not contribute to the reduction in synaptic release in this study.

## Discussion

In this study, we first examined the mutated APP expression and the A*β* secretion in the cultured cortical astrocytes from the Tg2576 mouse strain. APP is a type I transmembrane protein located in the cell membrane, *trans*‐Golgi network, endoplasmic reticulum, and endosomes. We found that mutant APP was dramatically overexpressed in cultured cortical astrocytes from Tg2576 mice. Literature shows that the astrocyte functions as a key player not only in the synaptic formation, maturation, and elimination, but also in the regulation of synaptic transmission (Giaume et al. 2010). Hence, abnormality in astrocytes should also be associated with the progressive synaptic degeneration in the AD brain (Rodriguez et al. 2009; Salmina 2009; Olabarria et al. 2010).

### Functional roles of astrocytes in synaptic transmission related to the overexpression of mutant APP

The literature suggests that *β*‐secretase immunoreactivity is observed in astrocytes around senile plaques in aged Tg2576 mice (Hong et al. 2003). Because Tg2576 mice possess mutant APP that is easily cleaved by *β*‐secretase, A*β* production may be progressively accelerated during the culture of Tg2576 astrocytes. Hence, we speculated that synaptic dysfunction could be explained by our result: the cultured Tg2576 astrocytes secreted higher concentrations of A*β* 1–40 and 1–42 compared with WT. (Fig. 1C). In our culture conditions, a WT neuron was chronically subjected to A*β* secreted from the Tg astrocytes during the synapse formation (>14 days at least in vitro). However, the average concentrations of A*β* 1–40 and 1–42 released from cultured Tg astrocytes are estimated to be 0.92 nmol/L and 0.05 nmol/L, respectively, which appears to be physiologically much lower than the amount that can elicit a significant reduction in synaptic transmission. It has been reported that, at least in the case of a 3‐day incubation with A*β*, a concentration of >0.05 *μ*mol/L is necessary to elicit a significant decrease in the synaptic release (Parodi et al. 2010). In line with this observation, in our study, neurons cocultured with Tg astrocytes never exhibited synaptic dysfunction during neuronal culture (Fig. 2). This could be explained as follows: concentrations of A*β* supplied from Tg astrocytes have been much lower than the concentration necessary for alteration of the synaptic transmission. In addition to this explanation, the average concentrations of A*β* 1–40 and 1–42 released from aged Tg astrocytes (Fig. 3C) were less than that for younger Tg astrocytes (Fig. 1C). This could also rule out the possibility that exogenous lower concentration of A*β* affect synaptic transmission in aged Tg astrocytes. Thus, our data suggest that the appearance of synaptic dysfunction depends on the stage of senescence, in addition to a certain A*β* concentration level and period of A*β* exposure.

Because glial cells have a critical role in A*β* clearance (Alarcon et al. 2005; Pihlaja et al. 2008), it is believed that astrocytes participate in the reduction in the neuronal aberrations caused by A*β*. In contrast to such a protective effect, toxic factors involved in nitrogen monoxide and interleukin pathways are also secreted from astrocytes activated by A*β* (Gitter et al. 1995; Hu et al. 1998). It would therefore be only natural that the physiological balance between the neuronal protective capacity of the astrocytes and the ongoing neuronal anomalies caused by the toxic factors has strong influences on the progression of AD with age. Such A*β* clearance capacity of astrocytes should be physiologically maintained in general. Therefore, we believe that our experimental conditions, that is, unaged Tg astrocytes, would be sufficient for protection from A*β*‐induced synaptic abnormalities.

### Abnormality of synaptic transmission as a result of a mutant APP that emerged excessively in the aged astrocyte culture

The amyloid hypothesis that A*β* causes the neuronal degeneration observed in AD has been supported widely, and there are many reports that both APP and A*β* modulate the synaptic transmission (Small et al. 2001; Selkoe 2002; Kamenetz et al. 2003). For instance, autaptic neuronal culture with APP knocked out exhibits enhancement of the synapse formation and function (Priller et al. 2006). On the other hand, autapse hippocampal culture that expresses APP excessively elicits presynaptic and postsynaptic dysfunction underlying lowered vesicle recycling and AMPA receptor‐mediated inactivation (Ting et al. 2007). The precise mechanisms underlying astrocytic APP‐induced synaptic modulation are poorly understood because most of the previous reports have focused on APP and A*β* of neuronal origin. We therefore took advantage of the autapse culture system that was technically composed of mutant APP‐overexpressing astrocytes and a single wild‐type neuron, which would be tightly attached to the astrocyte cushion. We found that an autaptic single WT hippocampal neuron cocultured with aged Tg2576 astrocytes significantly decreases the evoked EPSC amplitude (Fig. 4B), mEPSC frequency (Fig. 4E), and the number of synaptic vesicles in RRP (Fig. 5B) without any changes in the vesicular release probability (Fig. 5C).

We also demonstrated that the overexpression of astrocytic mutant APP decreases the number of synapses per neuron (Fig. 5F). The decrease in synapse number would support decreases in the miniature synaptic release frequency and the RRR size. In addition to this interpretation, it is possible that the relative number of synaptic vesicles per single synapse may be decreased. To test this possibility, further experiments with synaptic vesicles in a single synapse using an electron microscope and a cytological study of the protein localization would be useful. On the other hand, no change in the mEPSC amplitude indicates that the postsynaptic function was not affected by astrocytic overexpression of mutant APP. Similar phenotypes have been reported in neurons expressing mutant presenilin 1, which is considered to be the gene responsible for familial AD. This protein upregulates the A*β* 1–42 production (Priller et al. 2007). Such phenotypes have also been observed in neurons cocultured with aged WT astrocytes (Kawano et al. 2012).

## Conclusions

Neuronal APP directly affects the formation and function of the synapses (Priller et al. 2006). Thus, it is possible that the astrocytic APP mutation, at least in part, is involved in the synaptic dysfunction because the astrocyte creates a tripartite synapse. Therefore, we can conclude that the abnormality in synapse transmission is caused by mutant APP produced excessively in the aged astrocytes. Because the astrocytes are closely associated with neurons in the central nervous system, astrocytes are expected to influence the progressive cognitive deterioration in AD (Salmina 2009). Glial cells constitute a majority of cells in the central nervous system, and glial cells were recently reported to be closely involved in AD and other neurological diseases. Thus, the research of astrocytes could become a convenient and quantitative platform for the development of new therapeutic modalities.

## Ethical Approval

All procedures regarding animal care were performed in strict accordance with the rules of the Experimental Animal Care and Ethics Committee of Fukuoka University (comparable to the NIH guidelines), after approval of the experimental protocol (Permit Numbers: 1002364 and 1203540).

## Conflicts of Interest

The authors have no actual or potential conflicts of interest.
